# Tanshinone IIA Attenuates Pulmonary Fibrosis via Dual Inhibition of JNK and Smad Signaling

**DOI:** 10.3390/antiox15070836

**Published:** 2026-07-02

**Authors:** Congying Guo, Sheng Ai, Jun Chen

**Affiliations:** 1 State Key Laboratory of Natural Medicines, China Pharmaceutical University, Nanjing 210009, China; congyguo@stu.cpu.edu.cn (C.G.); aisheng@stu.cpu.edu.cn (S.A.); 2 Department of Pharmacognosy, School of Traditional Chinese Pharmacy, China Pharmaceutical University, Nanjing 210009, China

**Keywords:** tanshinone IIA, fibroblast activation, pulmonary fibrosis, NADPH oxidase 4, JNK and Smad signaling

## Abstract

This study investigated the mechanism of TGF-β1-induced Nox4 expression in pulmonary fibrosis (PF) and the anti-fibrotic effects of Tanshinone IIA (Tan-IIA). In a bleomycin-induced pulmonary fibrosis mouse model and in TGF-β1-stimulated fibroblasts, Tan-IIA attenuated fibrosis, oxidative stress, and fibroblast activation. Pharmacological inhibition revealed that the JNK/c-Jun and Smad3 pathways cooperatively mediate TGF-β1-induced expression of Nox4 and fibrotic markers (Collagen I/III, α-SMA). Tan-IIA exerted these effects by dually inhibiting the JNK/c-Jun and Smad2/3 pathways, reducing their phosphorylation and nuclear signaling, which consequently suppressed Nox4 transcription and protein expression. The combination of Tan-IIA with JNK or Smad3 inhibitors synergistically enhanced these effects. We identified a tandem c-Jun/Smad binding element in the Nox4 promoter that is critical for TGF-β1 response. Reporter assays and CUT&RUN experiments confirmed that TGF-β1-induced transcriptional activation depends on an intact c-Jun/Smad binding element and recruitment of c-Jun and Smad2/3. Moreover, Tan-IIA inhibited the enrichment of c-Jun and Smad2/3 at the Nox4 promoter. Collectively, our findings demonstrate that a c-Jun/Smad element integrates profibrotic JNK and Smad signaling to drive Nox4 expression. Tan-IIA presents a novel therapeutic strategy for fibrosis by simultaneously targeting these two key pathways, thereby mitigating Nox4-dependent oxidative stress and fibroblast activation.

## 1. Introduction

Pulmonary fibrosis (PF) is a progressive and lethal lung disorder characterized by the destruction of lung parenchymal structures [1,2,3]. This scarring results from the aberrant activation of fibroblasts into myofibroblasts, which secrete extracellular matrix (ECM) proteins, primarily collagens, leading ultimately to respiratory failure [4,5,6,7]. Current antifibrotic therapies provide only modest clinical benefits, and effective treatment strategies remain elusive. Moreover, the underlying molecular mechanisms of PF have yet to be fully elucidated. Novel agents that effectively target these mechanisms are urgently needed.

Transforming growth factor-β 1 (TGF-β1) is a key regulator of cell migration, proliferation, and differentiation, and plays a central role in the pathogenesis of organ fibrosis. TGF-β1 induces Smad phosphorylation. Phosphorylated Smad proteins translocate to the nucleus, where they regulate the transcription of genes such as NADPH oxidase 4 (Nox4), α-smooth muscle actin (α-SMA), and collagen. Diverse transcriptional responses to TGF-β also require c-Jun. Studies indicate that Smads and c-Jun act synergistically in response to TGF-β and function as key downstream effectors of TGF-β signaling [8,9,10,11,12]. Emerging evidence suggests that transcriptional responses to TGF-β1 often involve the coordinated action of Smad complexes with other transcription factors, such as c-Jun [13,14]. The JNK pathway is increasingly recognized as an important contributor to fibrogenesis [15,16]. Nox4 is a key downstream effector of TGF-β1 signaling. Its upregulation generates sustained oxidative stress, creating a microenvironment that promotes myofibroblast differentiation and perpetuates fibrosis [17,18]. Although the JNK and Smad pathways are both involved in TGF-β1 signaling and serve as important downstream effectors, the mechanisms by which they regulate Nox4 and oxidative stress remain unclear. Therefore, clarifying the relationship among JNK, Smad, and Nox4 is of great importance for understanding and combating oxidative stress.

The Nox4 promoter contains potential binding sites for Smad and c-Jun, suggesting the presence of an integrated regulatory element [19]. However, the existence and functional importance of a specific tandem c-Jun/Smad response element within the Nox4 promoter require definitive validation. Its role as a nexus for JNK/c-Jun and Smad2/3 cooperation, as well as its contribution to fibrotic gene expression, also remains to be established.

Natural products with multitarget potential, such as tanshinone IIA (Tan-IIA), a compound derived from the traditional Chinese herb *Salvia miltiorrhiza* Bunge, have shown antifibrotic promise [20,21]. Tan-IIA has been reported to modulate signaling pathways relevant to fibrosis [22]. However, whether Tan-IIA concurrently inhibits both JNK/c-Jun and Smad2/3 signaling to disrupt their cooperative regulation of Nox4 remains to be established.

We recently found a functional tandem c-Jun/Smad binding element in the murine Nox4 promoter that may be important for TGF-β1-induced Nox4 transcriptional activation. In this study, we investigated whether Tan-IIA modulates oxidative stress through the JNK/c-Jun and Smad2/3 pathways and whether its regulation of Nox4 is related to the c-Jun/Smad binding element. Our results showed that Tan-IIA significantly attenuated fibroblast activation both in vitro and in vivo by inhibiting the JNK/c-Jun and Smad2/3 pathways and downregulating Nox4. By inhibiting these two pathways, Tan-IIA regulates Nox4 expression and prevents PF-associated fibroblast activation.

## 2. Materials and Methods

### 2.1. Chemicals and Materials

Tanshinone IIA (Tan-IIA, purity 98%) was purchased from Shanghai Yuan Ye Biotechnology Co., Ltd. (Chengdu, China) and dissolved in dimethyl sulfoxide (DMSO; final concentration 0.1%, *v*/*v*). Recombinant human TGF-β1, anisomycin (a JNK activator), SIS_3_HCl (a Smad inhibitor) and SRI-011381 (a Smad activator) were obtained from Yeasen Biotechnology (Shanghai, China). SP600125 (a JNK inhibitor) and SB525334 (a TGF-β inhibitor) were obtained from MedChemExpress (Monmouth Junction, NJ, USA). Bleomycin (BLM) was purchased from Nippon Kayaku Co., Ltd. (Tokyo, Japan). The molecular structures of all compounds are shown in Supplementary Figure S1.

### 2.2. Cell Lines and Cell Culture

NIH-3T3 cells (a mouse embryonic fibroblast cell line) and MRC-5 cells (a human diploid lung fibroblast cell line) were obtained from the Cell Bank of the Chinese Academy of Sciences (Shanghai, China). NIH-3T3 cells were cultured in Dulbecco’s modified Eagle’s medium (DMEM; Gibco) supplemented with 10% fetal bovine serum (FBS; Yeasen Biotechnology, Shanghai, China) and penicillin–streptomycin (100 U/mL; Beyotime Institute of Biotechnology). MRC-5 cells were cultured in Minimum Essential Medium (MEM; Gibco) supplemented with 10% FBS, penicillin/streptomycin (100 U/mL), non-essential amino acids (NEAAs), sodium pyruvate, and glutamine (all from Gibco unless specified). All cells were maintained at 37 °C in a humidified incubator with 5% CO_2_.

### 2.3. CCK8 Assay

Cell viability was assessed using the Cell Counting Kit-8 (CCK-8; Vazyme, Nanjing, China) according to the manufacturer’s instructions. Cells were treated with Tan-IIA at (0, 0.1, 1, 10, 20, 30, 50 and 100 μM) for 24 h. Subsequently, 10 μL of CCK-8 solution was added to each well, and the plates were incubated at 37 °C for 1 h. All experiments were performed in biological triplicate. Absorbance was measured at 450 nm using a microplate reader (EnVision; Agilent Technologies, Santa Clara, CA, USA). Cytotoxicity was evaluated by normalizing absorbance values to those of untreated control cells.

### 2.4. Cell Treatment

Cells were seeded into 6-well plates. When cells reached approximately 80% confluence, they were treated as indicated in the experimental design. In the presence of 10 ng/mL TGF-β1, the culture medium was replaced with fresh medium containing either 0.1% DMSO or Tan-IIA (1, 5, or 10 μM), and the cells were incubated for 24 h. In the presence of 10 ng/mL TGF-β1, the medium was replaced with fresh medium containing 0.1% DMSO, SB525334 (a TGF-β receptor inhibitor, 10 μM), SP600125 (a JNK inhibitor, 30 μM), SIS_3_HCl (a Smad inhibitor, 10 μM), or a combination of SP600125 and SIS_3_HCl, followed by incubation for 24 h. In the presence of 10 ng/mL TGF-β1, the medium was replaced with fresh medium containing 0.1% DMSO, Tan-IIA (10 μM), SP600125, anisomycin (a JNK activator, 2 nM), SIS_3_HCl, SRI-011381 (a Smad activator, 15 μM), or Tan-IIA in combination with each of these compounds, followed by incubation for 24 h. separately. Cells were collected for subsequent analyses.

### 2.5. Determination of ROS Production

Intracellular reactive oxygen species (ROS) levels in NIH-3T3 cells were measured using the fluorescent probe 2′,7′-dichlorodihydrofluorescein diacetate (DCFH-DA). Cell nuclei were stained with Hoechst 33342 (Beyotime Institute of Biotechnology, Shanghai, China).

In the presence of 10 ng/mL TGF-β1, cells were incubated for 24 h in a medium containing 0.1% DMSO, Tan-IIA, SP600125, anisomycin, SIS_3_HCl, or SRI-011381. The medium was then replaced with serum-free medium containing DCFH-DA (10 μM), and the cells were incubated at 37 °C in the dark for 20 min. After three washes with PBS, fluorescence images were acquired using a confocal laser scanning microscope (Olympus FV3000, Tokyo, Japan).

### 2.6. Murine Model of BLM-Induced Lung Injury

Female-specific pathogen-free C57BL/6 mice (20 ± 2 g) were obtained from Vital River Laboratory Animal Technology Co., Ltd. (Hangzhou, China). Estrogen has been reported to promote fibrosis. Therefore, female mice were used because they provide a clearer pathological phenotype for antifibrotic drug screening. In addition, the use of female mice reduces behavioral issues such as male aggression, thereby improving experimental consistency [23]. The animals were housed under standard specific pathogen-free (SPF) conditions at 20–24 °C with 50 ± 10% relative humidity and a 12 h light/dark cycle, with ad libitum access to food and water. All animal procedures were conducted in accordance with the Chinese Experimental Animals Administration Legislation and were approved by the Animal Care and Use Committee of China Pharmaceutical University.

BLM (0.025 U) dissolved in saline was administered into the tracheal lumen using a needle, after which the incision was sutured. Sham-operated animals received an equivalent volume of saline. BLM-treated mice received 0.5% sodium carboxymethyl cellulose (CMC-Na) or Tan-IIA (10 or 20 mg/kg, dissolved in 0.5% CMC-Na), once daily for 21 consecutive days. Mice were deeply anesthetized by intraperitoneal injection of 1.25% tribromoethanol. Lung tissue was collected, after which the mice were euthanized by cervical dislocation. Lungs were excised and weighed. The right lobes were snap-frozen in liquid nitrogen and stored at −80 °C, whereas the left lobes were fixed in 4% buffered formalin.

### 2.7. Histology Analysis

Mouse lung tissues were fixed in 4% buffered formalin. The tissues were dehydrated, embedded in paraffin, sectioned at 6 μm, and stained with hematoxylin and eosin (H&E; Jiancheng Bioengineering Institute, Nanjing, China). For Masson’s trichrome staining, sections were deparaffinized, rehydrated, and stained using a commercial kit (Beyotime Institute of Biotechnology, Shanghai, China). In both cases, stained sections were scanned and processed using KFSlideOS software (version.1.0.5).

### 2.8. Quantitative Real-Time PCR (qRT-PCR)

Total RNA was extracted from cultured cells or lung tissue samples. RNA concentration and purity were determined using a spectrophotometer. First-strand cDNA was synthesized from 1 μg of total RNA using a commercial reverse transcription kit (Vazyme, Nanjing, China). The resulting cDNA was used immediately for qPCR or stored at −20 °C until use. Quantitative real-time PCR was performed using an ABI sequence detection system (Applied Biosystems, Waltham, MA, USA) to quantify gene expression. Relative expression levels were calculated via the comparative Ct (2^−ΔΔCt^) method, normalized to the housekeeping gene GAPDH. The analyzed targets included Col-I, Col-III, α-SMA, and Nox4 (Table 1).

### 2.9. Nox4 Promoter–Reporter Construction and Transfection

A 5.51 kb fragment of the mouse Nox4 promoter (ENSMUST00000032781.14) was cloned into the pGL3-Basic vector to generate the wild-type reporter plasmid (Nox-mus-5512 bp, wild type). Site-directed mutagenesis was then performed on this plasmid to mutate the c-Jun/Smad-binding element (TGAGTCACTGTCTGG). Specifically, the sixth, seventh, tenth, twelfth, and fourteenth nucleotides were altered to generate the sequence TGAGTTCCTCTATCG, yielding the mutant plasmid (Nox-mus-5512 bp mutant). All plasmids were constructed and synthesized by Genewiz (Nanjing, China). For transfection, NIH-3T3 cells in 96-well-seeded plates were transfected with 0.25 μg of plasmid DNA at a 9:1 ratio of reporter plasmid to β-galactosidase (β-gal) plasmid. DNA was mixed with Rfect Plasmid DNA Transfection Reagent (Puoneng Biotechnology, Nantong, China) according to the manufacturer’s instructions to form DNA–reagent complexes. A 20 μL aliquot of the complexes was added to each well, followed by incubation at 37 °C for 24 h. After incubation, the cells were harvested and lysed. Aliquots of the lysate were assayed for luciferase chemiluminescence and β-galactosidase activity.

### 2.10. CUT&RUN-qPCR

CUT&RUN was performed using the Hyperactive pG-MNase CUT&RUN Assay Kit (Vazyme, HD101, Nanjing, China) according to the manufacturer’s instructions. Briefly, 1 × 10^5^ fresh cells were washed and incubated overnight at 4 °C in an antibody buffer containing primary antibodies against p-Smad2/3, p-Jun, or control IgG. After two washes with Dig-wash buffer, the cells were incubated with pG-MNase for 1 h at 4 °C. Chromatin cleavage was initiated by adding a Dig-wash buffer supplemented with 2 mM CaCl_2_. Following a 1 h incubation on ice, the reaction was stopped. DNA fragments were released by incubation at 37 °C for 30 min. The supernatant containing the released DNA fragments was collected by centrifugation at 16,000× *g* for 5 min at 4 °C, and DNA was purified using the provided FastPure gDNA Mini Columns. Nox4 promoter enrichment was quantified by real-time PCR on an ABI Sequence Detection System, and the primers are listed in Table 1. Relative enrichment was calculated using the 2^−ΔΔCt^ method, with spike-in DNA for normalization control and the IgG sample as the calibrator.

### 2.11. Western Blotting

Total protein was extracted from cells and lung tissues, separated by SDS-PAGE, and transferred onto nitrocellulose membranes. The primary antibodies used were as follows: anti-collagen I (1:1000, 14695-1-AP) and anti-collagen III (1:1000, 22734-1-AP) from Proteintech (Wuhan, China); anti-α-SMA (1:1000, ET1607-53), anti-Nox4 (1:2000, ET1607-4), anti-phospho-JNK1/2/3 (Thr183/Tyr185) (1:3500, ET1609-42), anti-phospho-c-Jun (Ser63) (1:1000, ET1608-4), anti-JNK1/2/3 (1:3500, ET1601-28), anti-c-Jun (1:1000, ET1608-3), anti-phospho-Smad2 (Ser250) (1:1000, CY5608), anti-phospho-Smad3 (Ser423/425) (1:1000, HA750172), anti-Smad2 (1:1000, ET1604-22), anti-Smad3 (1:1000, ET1607-41), and anti-Smad4 (1:1000, ET1604-12) from Huabio (Hangzhou, China); and anti-GAPDH (1:5000, AB0037) from Abways Technology (Shanghai, China). After overnight incubation with the primary antibodies at 4 °C, the membranes were incubated with an HRP-conjugated goat anti-rabbit IgG (H+L) secondary antibody for 2 h. Protein bands were visualized and quantified using Gel-Pro 32 software.

### 2.12. Statistical Analysis

Quantitative data are presented as the mean ± standard deviation (SD). All statistical analyses were performed using GraphPad Prism 8 (GraphPad Software, San Diego, CA, USA). Differences between the two groups were assessed using a two-tailed Student’s *t*-test. Comparisons among three or more groups were performed using one-way analysis of variance (ANOVA), followed by Tukey’s post hoc test. A *p*-value < 0.05 was considered statistically significant.

## 3. Results

### 3.1. Tan-IIA Attenuates Pulmonary Fibrosis and Oxidative Stress

**Figure 1:**
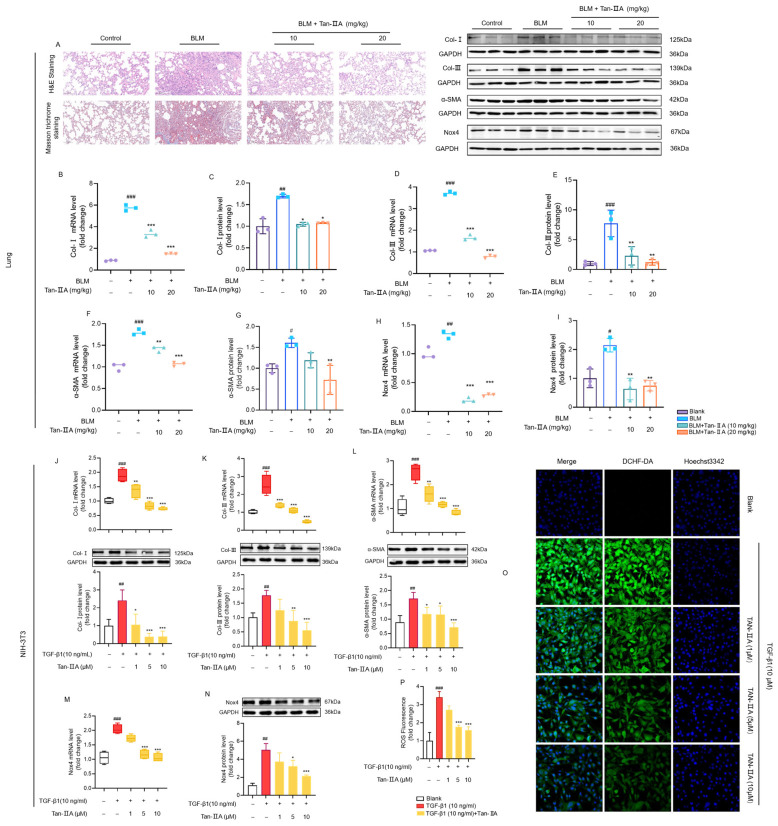
Tan-IIA attenuates pulmonary fibrosis and oxidative stress. (**A**) Representative photomicrographs of lung tissue sections stained with H&E and Masson’s trichrome. Scale bar = 100 μm. (**B**–**I**) mRNA and protein expression levels of Col-I, Col-III, α-SMA, and Nox4 in lung tissues from mice treated with BLM (0.025 U) and Tan-IIA. (**J**–**N**) mRNA and protein expression levels of Col-I, Col-III, α-SMA, and Nox4 in NIH-3T3 cells treated with TGF-β1 (10 ng/mL) and Tan-IIA. (**O**,**P**) Representative confocal images and quantification of ROS levels in NIH-3T3 cells after the indicated treatments. Scale bar = 100 μm. −/+ indicates absence or presence of compound treatment, respectively. Data are expressed as the mean ± SD. Experiments were repeated three times. # *p* < 0.05, ## *p* < 0.01, ### *p* < 0.001 vs. Control; * *p* < 0.05, ** *p* < 0.01, *** *p* < 0.001 vs. BLM or TGF-β1.

In vivo: BLM administration in mice is a widely used model of pulmonary fibrosis that recapitulates key clinicopathological features of idiopathic pulmonary fibrosis (IPF). We established a BLM-induced model in C57BL/6J mice to investigate the effects of Tan-IIA on pulmonary fibrosis (PF). After Tan-IIA treatment, lung tissues from each group were photographed under bright-field microscopy. Compared with the sham group, lung tissues from the BLM group showed marked pathological alterations. These alterations were markedly alleviated by Tan-IIA treatment (Figure 1A). Tan-IIA significantly suppressed the BLM-induced upregulation of Col-I, Col-III, α-SMA, and Nox4 at both the mRNA and protein levels (Figure 1B–I).

In vitro: Tan-IIA exhibited no cytotoxicity at concentrations ranging from 1 μM to 10 μM in NIH-3T3 and MRC-5 cells. This finding excluded the possibility that the inhibitory effect of Tan-IIA on fibroblast activation was due to cytotoxicity (Supplementary Figure S2). Further experiments using TGF-β1-induced fibroblast activation demonstrated that Tan-IIA reduced the mRNA and protein levels of Col-I, Col-III, α-SMA, and Nox4 in a concentration-dependent manner (Figure 1J–N). Similarly, Tan-IIA also reduced the mRNA and protein levels of Col-I, Col-III, α-SMA, and Nox4 in MRC-5 cells (Supplementary Figure S3). TGF-β1 treatment markedly increased ROS-associated green fluorescence, whereas Tan-IIA significantly attenuated this increase in a concentration-dependent manner (Figure 1O,P). Collectively, these findings indicate that Tan-IIA suppresses the expression of extracellular matrix components and myofibroblast markers in BLM-induced pulmonary fibrosis. Tan-IIA also suppressed TGF-β1-induced fibrosis-related protein expression and intracellular oxidative stress in NIH-3T3 cells.

### 3.2. Tan-IIA Suppresses Pulmonary Fibrosis by Inhibiting JNK/c-Jun and Smad2/3 Signaling

**Figure 2:**
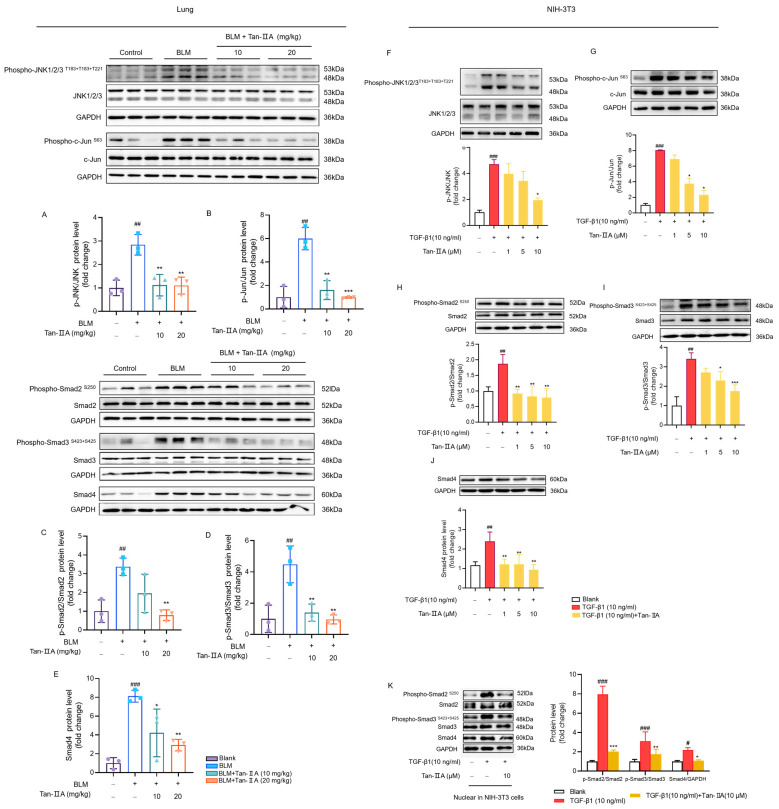
Tan-IIA attenuates pulmonary fibrosis by inhibiting activation of the JNK/c-Jun and Smad2/3 pathways and reducing Nox4 expression. (**A**–**E**) Protein levels of p-JNK/JNK, p-c-Jun/c-Jun, p-Smad2/Smad2, p-Smad3/Smad3, and Smad4 in lung tissues from mice treated with BLM and Tan-IIA. (**F**–**J**) Protein levels of p-JNK/JNK, p-c-Jun/c-Jun, p-Smad2/Smad2, p-Smad3/Smad3, and Smad4 in NIH-3T3 cells treated with TGF-β1 and Tan-IIA. (**K**) Nuclear levels of p-Smad2/Smad2, p-Smad3/Smad3, and Smad4 after the indicated treatments. −/+ indicates absence or presence of compound treatment, respectively. Data are expressed as the mean ± SD. The experiment was repeated three times. # *p* < 0.05, ## *p* < 0.01, ### *p* < 0.001 vs. Control; * *p* < 0.05, ** *p* < 0.01, *** *p* < 0.001 vs. BLM or TGF-β1.

The expression of JNK and Smad pathway-related proteins was examined in lung tissues from the mouse pulmonary fibrosis model. In vivo, sustained Tan-IIA administration significantly reduced BLM-induced phosphorylation of JNK, c-Jun, and Smad2/3, as well as Smad4 expression (Figure 2A–E). Consistent with these findings, in vitro experiments in TGF-β1-treated NIH-3T3 cells showed that Tan-IIA similarly reduced the phosphorylation of JNK, c-Jun, and Smad2/3, as well as Smad4 expression, in a concentration-dependent manner (Figure 2F–J). Phosphorylated Smad2/3 forms a complex with Smad4 and translocates from the cytoplasm to the nucleus, where it regulates gene transcription [24]. Tan-IIA significantly reduced the nuclear levels of p-Smad2/3 and Smad4 (Figure 2K). Collectively, these results demonstrate that Tan-IIA attenuates fibrosis by suppressing the JNK and Smad signaling pathways, thereby inhibiting Nox4 expression both in vivo and in vitro.

### 3.3. Combined Inhibition of c-Jun and Smad3 Synergistically Reduces Fibrogenic Gene Expression in Fibroblasts

**Figure 3:**
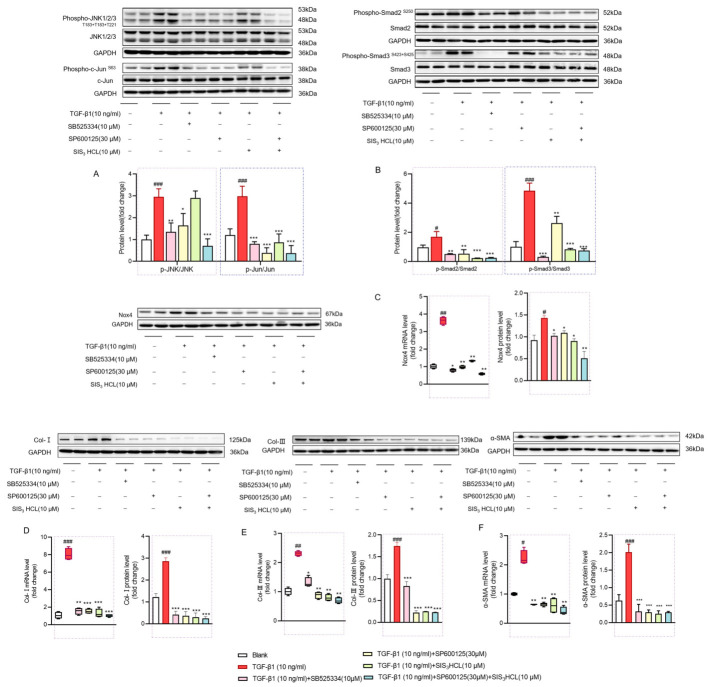
Inhibition of JNK and Smad signaling attenuates TGF-β1-induced expression of Nox4 and fibrosis-related markers. (**A**) Protein levels of p-JNK/JNK and p-c-Jun/c-Jun in NIH-3T3 cells after the indicated treatments. (**B**) Protein levels of p-Smad2/Smad2 and p-Smad3/Smad3 in NIH-3T3 cells after the indicated treatments. (**C**–**F**) mRNA levels (*n* = 4) and protein expression (*n* = 3) of Nox4, Col-I, Col-III, and α-SMA in NIH-3T3 cells after the indicated treatments. −/+ indicates absence or presence of compound treatment, respectively. Data are presented as the mean ± SD. Experiments were repeated three times. # *p* < 0.05, ## *p* < 0.01, ### *p* < 0.001 vs. Control; * *p* < 0.05, ** *p* < 0.01, *** *p* < 0.001 vs. TGF-β1.

To further verify that c-Jun and Smad cooperate to regulate Nox4 expression and oxidative stress, TGF-β1-stimulated NIH-3T3 cells were treated with the ALK5 (TGF-β receptor) inhibitor SB525334, the JNK inhibitor SP600125, or the Smad inhibitor SIS_3_HCl in the absence of Tan-IIA (Figure 3). Blocking TGF-β signaling simultaneously reduced the activation of both JNK and Smad pathways (Figure 3A,B). Furthermore, inhibition of TGF-β signaling, JNK, Smad, or both JNK and Smad reduced Nox4 mRNA and protein expression, suggesting that both c-Jun and Smad2/3 are required for Nox4 regulation (Figure 3C).

To determine whether c-Jun and Smad also contribute to antifibrotic effects, we performed the same inhibitor treatments. We found that inhibition of TGF-β signaling, JNK, Smad, or both JNK and Smad together (SP600125 + SIS_3_HCl) markedly decreased the mRNA and protein levels of Col-I, Col-III, and α-SMA (Figure 3D–F). In MRC-5 cells, the results were consistent with NIH-3T3 (Supplementary Figure S4). These results indicate that inhibition of c-Jun and Smad2/3 attenuates fibroblast activation and that both transcription factors act downstream of TGF-β signaling.

### 3.4. Tan-IIA Targets Both JNK and Smad Pathways to Suppress Nox4-Driven Oxidative Stress

**Figure 4:**
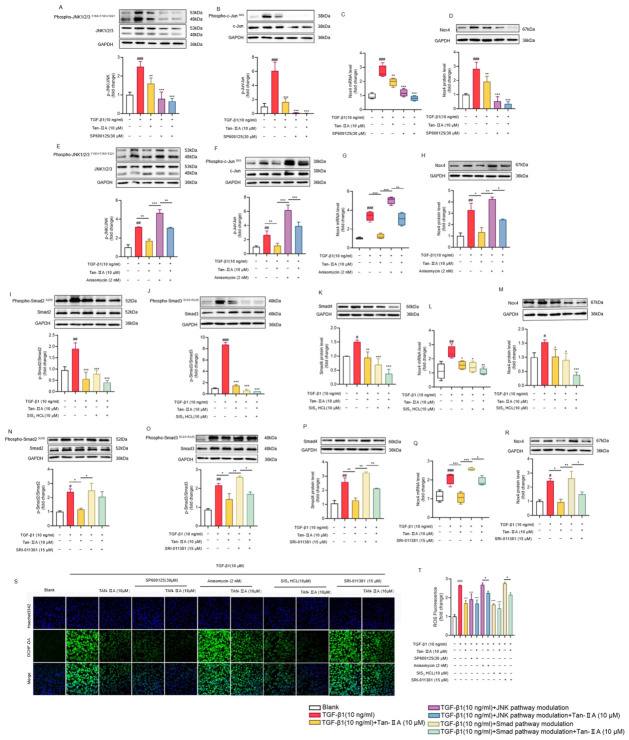
Tan-IIA suppresses JNK and Smad signaling, thereby attenuating Nox4 expression and ROS generation. (**A**–**D**) Protein levels (*n* = 3) of p-JNK/JNK, p-c-Jun/c-Jun, and Nox4, as well as Nox4 mRNA expression, in cells treated with Tan-IIA and SP600125 (30 μM). (**E**–**H**) Protein levels of p-JNK/JNK, p-c-Jun/c-Jun, and Nox4, as well as Nox4 mRNA expression, in cells treated with Tan-IIA and anisomycin (2 nM). (**I**–**M**) Protein levels of p-Smad2/Smad2, p-Smad3/Smad3, Smad4, and Nox4, as well as Nox4 mRNA expression, in cells treated with Tan-IIA and SIS_3_HCl (10 μM). (**N**–**R**) Protein levels of p-Smad2/Smad2, p-Smad3/Smad3, Smad4, and Nox4, as well as Nox4 mRNA expression, in cells treated with Tan-IIA and SRI-011381 (15 μM). (**S**,**T**) Representative confocal images and quantification of ROS levels in NIH-3T3 cells after the indicated treatments. Scale bar = 100 μm. −/+ indicates absence or presence of compound treatment, respectively. Data are expressed as the mean ± SD. The experiment was repeated three times. # *p* < 0.05, ## *p* < 0.01, ### *p* < 0.001 vs. Control; * *p* < 0.05, ** *p* < 0.01, *** *p* < 0.001 vs. TGF-β1.

To further investigate how Tan-IIA regulates Nox4 expression through the JNK and Smad pathways, NIH-3T3 cells were stimulated with TGF-β1. TGF-β1 stimulation increased the phosphorylation of JNK, c-Jun, and Smad2/3, and upregulated Smad4 protein expression levels. TGF-β1 significantly increased Nox4 mRNA and protein expression. Tan-IIA significantly inhibited TGF-β1-induced phosphorylation of JNK, c-Jun, and Smad2/3, and reduced Smad4 and Nox4 expression (Figure 4).

Inhibition of JNK signaling with SP600125 markedly suppressed Nox4 expression at both the mRNA and protein levels in NIH-3T3 cells (Figure 4A–D). Conversely, activation of JNK signaling by anisomycin significantly increased Nox4 expression at both the mRNA and protein levels (Figure 4E–H). Treatment with the Smad inhibitor SIS_3_HCl reduced Smad4 protein levels and decreased Nox4 expression at both the mRNA and protein levels (Figure 4I–M). In contrast, activation of Smad signaling by SRI-011381 upregulated Smad4 and Nox4 expression (Figure 4N–R). Moreover, Tan-IIA in combination with the JNK inhibitor SP600125 or the Smad inhibitor SIS_3_HCl further enhanced the inhibitory effect on Nox4 expression. When Tan-IIA was combined with the JNK activator anisomycin or the Smad activator SRI-011381, the stimulatory effects of these activators on the indicated molecules were reversed (Figure 4). These results indicate that Tan-IIA suppresses Nox4 expression through inhibition of both the JNK and Smad pathways.

Intracellular reactive ROS levels were measured using DCFH-DA in TGF-β1-stimulated NIH-3T3 cells treated with Tan-IIA, SP600125, anisomycin, SIS_3_HCl, or SRI-011381, either alone or in combination. Tan-IIA, SP600125, and SIS_3_HCl, either alone or in combination, reduced the TGF-β1-induced increase in ROS. In contrast, activation of the JNK or Smad pathway increased ROS-associated fluorescence, whereas Tan-IIA inhibited the anisomycin- or SRI-011381-induced increase in ROS (Figure 4S,T).

### 3.5. Tan-IIA Suppresses Myofibroblast Activation by Dual Inhibition of JNK and Smad Signaling

**Figure 5:**
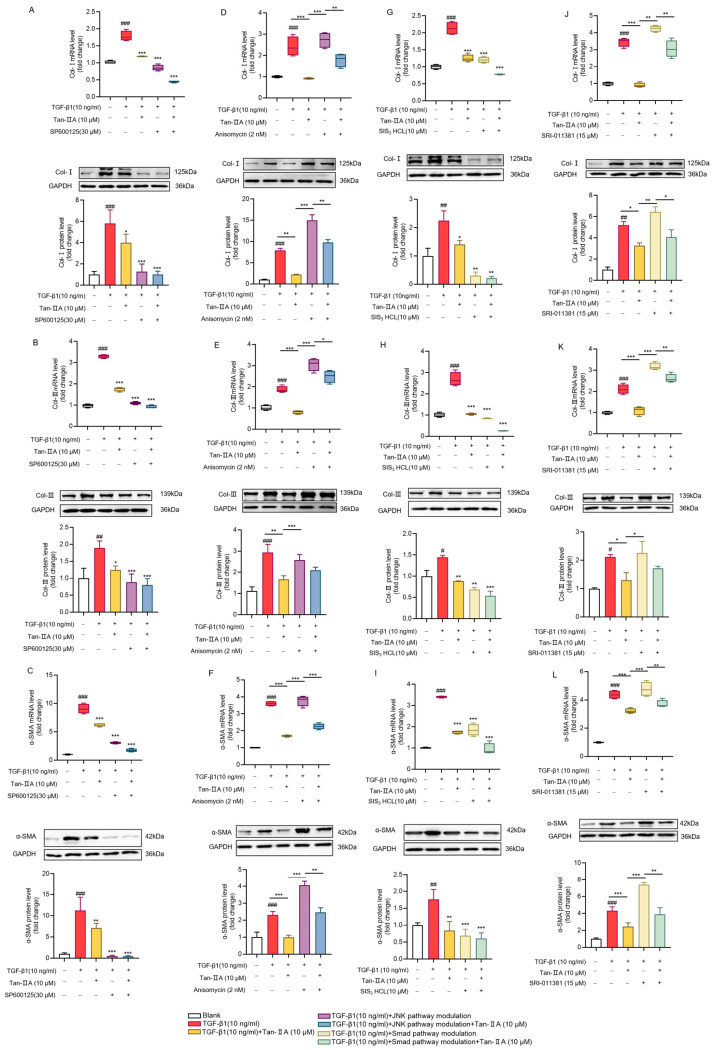
Tan-IIA attenuates TGF-β1-induced myofibroblast activation through dual inhibition of JNK and Smad signaling. (**A**–**C**) mRNA and protein expression levels of Col-I, Col-III, and α-SMA in NIH-3T3 cells treated with Tan-IIA and SP600125. (**D**–**F**) mRNA and protein expression levels of Col-I, Col-III, and α-SMA in NIH-3T3 cells treated with Tan-IIA and anisomycin. (**G**–**I**) mRNA and protein expression levels of Col-I, Col-III, and α-SMA in NIH-3T3 cells treated with Tan-IIA and SIS_3_HCl. (**J**–**L**) mRNA and protein expression levels of Col-I, Col-III, and α-SMA in NIH-3T3 cells treated with Tan-IIA and SRI-011381. Data are expressed as the mean ± SD. The experiment was repeated three times. # *p* < 0.05, ## *p* < 0.01, ### *p* < 0.001 vs. Control; * *p* < 0.05, ** *p* < 0.01, *** *p* < 0.001 vs. TGF-β1.

To investigate the roles of the JNK and Smad pathways in fibroblast activation, we evaluated fibroblast marker expression after inhibition or activation of these pathways. In TGF-β1-induced NIH-3T3 cells, Tan-IIA treatment or JNK inhibition with SP600125 significantly reduced the mRNA and protein levels of Col-I, Col-III, and α-SMA (Figure 5A–C). Activation of JNK with anisomycin increased the mRNA and protein levels of Col-I, Col-III, and α-SMA. However, co-treatment with anisomycin and Tan-IIA reversed the anisomycin-induced upregulation of these markers (Figure 5D–F).

Similarly, treatment with the Smad inhibitor SIS_3_HCl reduced the mRNA and protein levels of Col-I, Col-III, and α-SMA in TGF-β1-induced NIH-3T3 cells. The combination of SIS_3_HCl and Tan-IIA exerted a stronger inhibitory effect (Figure 5G–I). Conversely, treatment with the Smad activator SRI-011381 alone increased Col-I, Col-III, and α-SMA expression in NIH-3T3 cells. Co-treatment with SRI-011381 and Tan-IIA reduced the expression of Col-I, Col-III, and α-SMA (Figure 5J–L). These results indicate that Tan-IIA effectively downregulates TGF-β1-induced myofibroblast marker expression by inhibiting the JNK and Smad signaling pathways, supporting its potential therapeutic role in pulmonary fibrosis.

### 3.6. An Intact c-Jun/Smad Element Is Required for TGF-β1-Induced Nox4 Transcription

**Figure 6:**
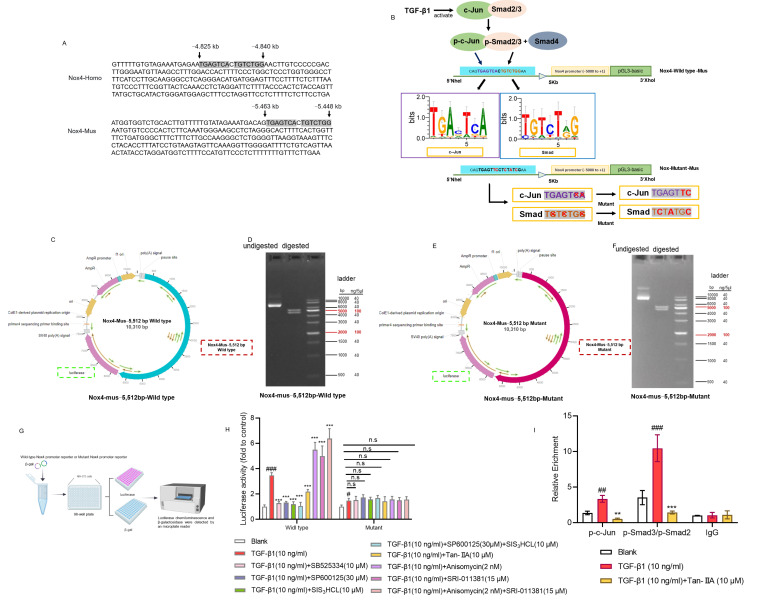
The c-Jun/Smad-binding element is essential for TGF-β1-induced transcriptional activation of the Nox4 promoter. (**A**) Location of the c-Jun/Smad-binding element in the human Nox4 promoter (NM_016931.5) and the murine Nox4 promoter. (**B**) Nucleotide sequence of the c-Jun/Smad-binding element. (**C**) Wild-type Nox4 promoter reporter construct. (**D**) Identification of the wild-type Nox4 promoter reporter construct by agarose gel electrophoresis. (**E**) Mutant Nox4 promoter reporter construct. (**F**) Identification of the mutant Nox4 promoter reporter construct by agarose gel electrophoresis. (**G**) Schematic diagram of the fluorescence detection method. (**H**) Fluorescence intensity of the two plasmids after the indicated drug treatments. (**I**) Tan-IIA treatment suppressed c-Jun and Smad2/3 enrichment at the Nox4 promoter. Data are expressed as the mean ± SD. The experiment was repeated three times. # *p* < 0.05, ## *p* < 0.01, ### *p* < 0.001 vs. Control; ** *p* < 0.01, *** *p* < 0.001 vs. TGF-β1. n.s., not significant.

Both the human and mouse Nox4 promoters contain a c-Jun/Smad element, located at approximately −4.83 kb in the human promoter and between −5.46 and −5.44 kb in the mouse promoter (Figure 6A) [19]. Previous studies have shown that the integrity of this c-Jun/Smad element in the human Nox4 promoter is critical for TGF-β1-mediated Nox4 activation [19]. The c-Jun and Smad binding sites are arranged in tandem and separated by only one base pair. To evaluate the requirement of the c-Jun/Smad element for TGF-β1-induced activity of the mouse Nox4 promoter and to investigate the modulatory effect of Tan-IIA on this element, a 5512 bp fragment upstream of the Nox4 transcription start site was cloned into the pGL3-Basic vector to generate the wild-type Nox4 promoter–reporter construct (Nox4-mus-5512 bp, wild type). The c-Jun site (2 bp) and Smad site (3 bp) within the c-Jun/Smad element were mutated by site-directed mutagenesis, and the resulting 5512 bp fragment was inserted into pGL3-Basic to generate the mutant Nox4 promoter–reporter construct (Nox4-mus-5512 bp, mutant) (Figure 6B,C,E). Both plasmids were verified by agarose gel electrophoresis. Electrophoretic analysis confirmed the presence of the 5512 bp Nox4 promoter fragment and the 4,818 bp pGL3-Basic vector backbone, indicating correct plasmid assembly and adequate purity (Figure 6D,F).

After transfection of the two constructs into NIH-3T3 cells, luciferase activity was measured and normalized to β-galactosidase (β-gal) activity. The wild-type Nox4 promoter–reporter construct showed a strong response to TGF-β1 stimulation compared with the control. This response was markedly suppressed by SB525334, SP600125, SIS3HCl, and Tan-IIA. In contrast, the mutant Nox4 promoter–reporter construct showed a significantly attenuated response to TGF-β1, and its activity was not affected by any of these inhibitors. Moreover, compared with the mutant construct, the wild-type Nox4 promoter–reporter plasmid showed a significantly enhanced response to TGF-β1-induced promoter activation after treatment with the JNK activator anisomycin or the Smad activator SRI-011381, whereas the mutant construct again showed no notable change (Figure 6G,H). CUT&RUN assays showed that TGF-β1 treatment significantly increased the enrichment of c-Jun and Smad2/3 at the c-Jun/Smad element compared with the control, whereas Tan-IIA suppressed this enrichment (Figure 6I). These results suggest that an intact c-Jun/Smad element in the Nox4 promoter is required for TGF-β1 responsiveness and that its function depends on c-Jun and Smad2/3 activity. Tan-IIA likely inhibits downstream signaling by interfering with the binding of c-Jun and Smad2/3 to the c-Jun/Smad element.

## 4. Discussion

Pulmonary fibrosis (PF) is a chronic, progressive interstitial lung disease caused by various forms of lung injury, including drug toxicity and infection [25,26]. This condition is characterized by excessive extracellular matrix (ECM) deposition, which disrupts normal lung architecture and leads to progressive functional decline [27,28]. As the primary organ for gas exchange, the lung exists in a highly oxidative environment and has evolved robust antioxidant defenses [29,30]. Disruption of these defenses causes oxidative stress, which leads to alveolar epithelial cell injury, fibroblast proliferation and differentiation into myofibroblasts, and excessive ECM deposition, thereby driving fibrotic progression [31,32,33]. NADPH oxidase 4 (Nox4), a major ROS-producing enzyme, has been implicated in the pathogenesis of numerous diseases [34,35,36]. Tan-IIA, a major bioactive compound derived from *Salvia miltiorrhiza*, has been shown to inhibit ECM deposition and ameliorate fibrosis in various organs [21,37,38,39]. Here, we demonstrate that Tan-IIA attenuates pulmonary fibrosis by suppressing myofibroblast activation and ECM deposition. This effect is mediated by dual inhibition of the JNK/c-Jun and Smad2/3 signaling pathways, which downregulates Nox4 expression and reduces oxidative stress. Our findings reveal a novel cooperative mechanism in which tandem c-Jun/Smad-binding elements within the Nox4 promoter integrate profibrotic signals, highlighting Tan-IIA as a multitarget therapeutic candidate for fibrosis.

BLM is widely used to establish experimental models of PF. It induces cellular injury and oxidative stress, which in turn prompt damaged epithelial cells to release TGF-β1. TGF-β1 is widely regarded as a key driver of fibrosis. Under TGF-β1 stimulation, pulmonary fibroblasts become activated and differentiate into myofibroblasts. These activated myofibroblasts produce collagen, thereby exacerbating PF [40,41]. We used a BLM-induced mouse model to investigate the effects of Tan-IIA on PF in vivo. Tan-IIA significantly ameliorated BLM-induced lung injury and inhibited the upregulation of Col-I, Col-III, α-SMA, and Nox4 at both the mRNA and protein levels in BLM-treated mice. In vitro, Tan-IIA attenuated TGF-β1-induced ROS generation in a concentration-dependent manner and reduced fibrosis-related protein expression and intracellular oxidative stress.

Smad proteins are a family of transcription factors that act as downstream mediators of TGF-β signaling. They mediate signaling induced by TGF-β and related ligands. Upon activation by TGF-β receptors, Smad proteins form complexes with Smad4. In the canonical TGF-β/Smad signaling model, Smad4 is generally considered a constitutively expressed cofactor whose total protein level remains relatively stable. However, emerging evidence suggests that Smad4 protein abundance is not static, but is tightly regulated by the ubiquitin-proteasome pathway. For example, the deubiquitinase USP13 has been reported to reverse Smad4 polyubiquitination, thereby preventing its proteasomal degradation and increasing total Smad4 protein levels without altering its transcriptional activity [42,43,44]. In our in vivo and in vitro models, TGF-β1 significantly increased Smad4 protein levels in bleomycin-treated mice and NIH-3T3 cells. We propose that this observation does not contradict the classical paradigm. Rather, it may reflect a non-canonical post-translational mechanism. Specifically, TGF-β1 may induce the expression or activity of USP13 or related deubiquitinases, thereby enhancing Smad4 stability, prolonging its half-life, and increasing total protein accumulation. This interpretation is consistent with recent reports describing a TGF-β1/USP13/Smad4 regulatory axis in lung fibroblasts.

Previous studies have shown that the Fos–Jun complex AP-1 binds to sites within the TGF-β promoter. c-Jun responds rapidly to TGF-β stimulation, with its mRNA being rapidly induced. Smads can also interact with AP-1. In reporter assays using a specific region of the PAI-1 promoter, the Smad3/4-binding site “GTCTAGAC” is required for the transcriptional response to TGF-β [45,46,47]. Tan-IIA significantly reduced BLM-induced or TGF-β1-induced phosphorylation of JNK, c-Jun, and Smad2/3, as well as Smad4 expression, in vivo and in vitro. Collectively, these results suggest that Tan-IIA attenuates fibrosis by inhibiting JNK and Smad signaling, thereby suppressing Nox4 expression.

Nox4, a major enzymatic source of ROS, is a well-established contributor to fibrogenesis [48,49,50]. Its upregulation promotes ROS production, leading to oxidative stress, fibroblast-to-myofibroblast differentiation, and ECM deposition. JNK and Smad are key downstream effectors of the central profibrotic mediator TGF-β1, and both participate in the regulation of cellular responses to oxidative stress [51]. To investigate the regulatory relationship among c-Jun, Smad, and Nox4, and to determine whether Tan-IIA inhibits Nox4 through these pathways to exert antioxidant effects, in NIH-3T3 fibroblasts, Tan-IIA significantly inhibited TGF-β1-induced phosphorylation of JNK, c-Jun, and Smad2/3, as well as Smad4 and Nox4 expression. Pharmacological inhibition of JNK with SP600125 or Smad with SIS_3_HCl suppressed Nox4 expression at both the mRNA and protein levels, and these effects were enhanced by co-treatment with Tan-IIA. Conversely, activation of JNK with anisomycin or Smad with SRI-011381 upregulated Nox4 expression, and this effect was reversed by co-treatment with Tan-IIA. ROS levels, measured by DCFH-DA fluorescence, paralleled Nox4 expression. Tan-IIA, SP600125, and SIS_3_HCl each reduced TGF-β1-induced ROS, and the inhibitory effect was greater when these agents were combined. Activation of JNK or Smad increased ROS levels, and this effect was attenuated by Tan-IIA. This regulation was accompanied by reduced myofibroblast activation. Tan-IIA, alone or combined with SP600125 or SIS_3_HCl, inhibited TGF-β1-induced expression of Col-I, Col-III, and α-SMA. Activation of JNK or Smad upregulated these markers, and this effect was counteracted by Tan-IIA. Further mechanistic studies confirmed that Tan-IIA downregulates Nox4 by inhibiting the JNK and Smad pathways.

The importance of the c-Jun/Smad-binding element was first identified in the human Nox4 promoter, and we also detected a similar sequence in the murine Nox4 promoter [19]. To test this hypothesis, a 5512 bp fragment of the murine Nox4 promoter was cloned into the pGL3-Basic vector to generate two reporter constructs: a wild-type construct and a mutant construct carrying a disrupted c-Jun/Smad element (TGAGTCACTGTCTGG mutated to TGAGTTCCTATGTAG). The wild-type promoter showed a strong response to TGF-β1 stimulation. In addition, TGF-β1-induced promoter activation was suppressed by SB525334, SP600125, SIS_3_HCl, and Tan-IIA. Conversely, anisomycin and SRI-011381 further enhanced TGF-β1-induced promoter activity. The mutant promoter retained partial responsiveness to TGF-β1. However, its activity was markedly lower than that of the wild-type promoter. Moreover, treatment with these inhibitors, Tan-IIA, or the activators did not significantly alter luciferase activity in cells expressing the mutant construct. These results indicate that the c-Jun/Smad element is essential for the full responsiveness of the Nox4 promoter to TGF-β1 and that Tan-IIA modulates Nox4 transcription through this element. CUT&RUN analysis confirmed that, compared with the control group, TGF-β1 treatment significantly increased c-Jun and Smad2/3 enrichment at the Nox4 promoter, whereas Tan-IIA effectively inhibited this enrichment.

Natural products offer advantages over synthetic compounds in terms of biocompatibility and safety and are widely used in medicine and food-related applications. In recent years, research on natural products has advanced considerably, and the associated methods and technologies have become more rigorous and comprehensive [52,53]. Tan-IIA, a multitarget natural compound derived from the traditional Chinese medicinal herb *Salvia miltiorrhiza*, exhibits fewer toxic side effects than many conventional inhibitors. This study demonstrates that Tan-IIA attenuates pulmonary fibrosis by inhibiting the JNK/c-Jun and Smad2/3 signaling pathways. This effect reduces Nox4 expression and oxidative stress, thereby suppressing myofibroblast activation and extracellular matrix deposition. A conserved c-Jun/Smad-binding element in the Nox4 promoter integrates profibrotic signals and regulates transcription in response to TGF-β1. These findings elucidate a novel antifibrotic mechanism of Tan-IIA: inhibition of JNK and Smad signaling downregulates Nox4, reduces oxidative stress, and suppresses myofibroblast activation. This positions Tan-IIA as a potential therapeutic for pulmonary fibrosis via targeting the JNK/Smad-Nox4-ROS axis. Although our findings support a protective role for Tan-IIA in PF through coordinated inhibition of JNK/c-Jun and Smad2/3 signaling and subsequent suppression of Nox4, several limitations should be acknowledged. The mechanistic evidence was derived mainly from murine NIH-3T3 cells and a prophylactic BLM model. The effects of Tan-IIA have not yet been validated in clinical specimens or therapeutic intervention models. Therefore, caution is warranted when translating these findings into clinical practice, and further studies are needed to confirm their broader relevance and therapeutic potential.

## 5. Conclusions

**Figure 7:**
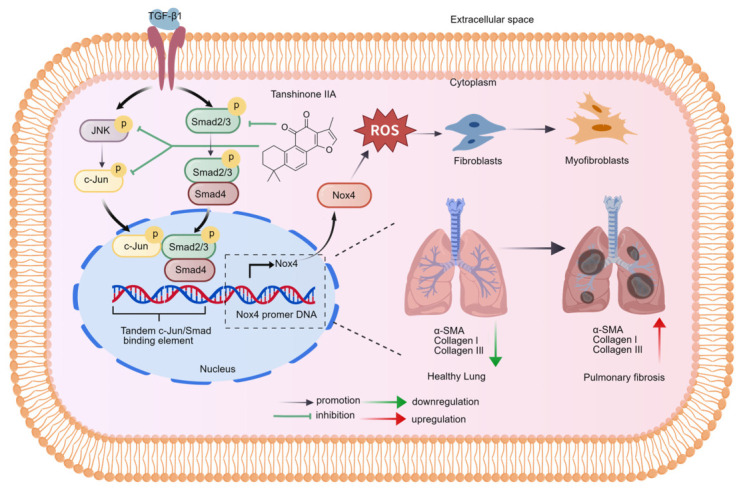
Tan-IIA suppresses pulmonary fibrosis by inhibiting JNK and Smad signaling. Tan-IIA exerts antifibrotic effects primarily through coordinated inhibition of JNK and Smad signaling, thereby reducing oxidative stress and fibrotic gene expression (Created with BioGDP.com).

In conclusion, our findings establish Tan-IIA as a potent inhibitor of pulmonary fibrogenesis, acting primarily through the suppression of Nox4-driven oxidative stress. We demonstrated that Tan-IIA mitigates bleomycin-induced structural deterioration in the lung while concurrently downregulating key fibrotic markers. Mechanistically, Tan-IIA antagonizes the TGF-β1 signaling axis through the coordinated inhibition of both the JNK/c-Jun and Smad2/3 pathways. Importantly, we identified a conserved c-Jun/Smad composite element within the Nox4 promoter that serves as a critical integration node for these profibrotic signals (Figure 7). Our data indicate that the c-Jun/Smad element in the Nox4 promoter serves as the core cis-acting element integrating signals from the JNK and Smad pathways. Tan-IIA therefore achieves precise transcriptional regulation of Nox4 by coordinately inhibiting these two pathways.

## Figures and Tables

**Table 1:** Primers used for qRT-PCR amplification.

Specifies	Gene Name	Sequence of Forward and Reverse Primers (5′ to 3′)	Accession Number
*Mus musculus*	*GAPDH*	Forward sequence: AGGTCGGTGTGAACGGATTTG	NM_001289726.2
		Reverse sequence: TGTAGACCATGTAGTTGAGGTCA	
	*Col-I*	Forward sequence: TTCTGTGGGTCCTGCTGGGAAA	NM_007742.4
		Reverse sequence: TTGTCACCTCGGATGCCTTGAG	
	*Col-* *III*	Forward sequence: CCCACATCACAGACTAACGAAT	NM_009930.2
		Reverse sequence: CGAAATCAAAGAAACCAGAACC	
	*α-SMA*	Forward sequence: GAAGCTCGTTATAGAAAGAGTGG	NM_007392.3
		Reverse sequence: TCAGGGAGTAATGGTTGGAAT	
	*Nox4*	Forward sequence: CAATCTTCTTGTTCTCCTGCTAG	NM_015760.5
		Reverse sequence: CATCCTTTTACCTATGTGCCG	
	*Nox4 CUT&RUN*	Forward sequence: TGATTCCATCAGGAGCTGCC	ENSMUST00000032781.14
		Reverse sequence: CTTAACCCCAGAGCCCTTGG	
*Homo sapiens*	*GAPDH*	Forward sequence: GAGTCCACTGGCGTCTTCA	NM_001256799.3
		Reverse sequence: GGGGTGCTAAGCAGTTGGT	
	*Col-I*	Forward sequence: GAGGGCCAAGACGAAGACATC	NM_000089.4
		Reverse sequence: CAGATCACGTCATCGCACAAC	
	*Col-* *III*	Forward sequence: GGAGCTGGCTACTTCTCGC	NM_000090.4
		Reverse sequence: GGGAACATCCTCCTTCAACAG	
	*α-SMA*	Forward sequence: CCTTGAGAAGAGTTACGAGTTGC	NM_001320855.2
		Reverse sequence: ATGATGCTGTTGTAGGTGGTTT	
	*Nox4*	Forward sequence: GCCAGAGTATCACTACCTCCAC	NM_016931.5
		Reverse sequence: CTCGGAGGTAAGCCAAGAGTGT	

## Data Availability

The data supporting the findings of this study are available within the article and its Supplementary Materials. Further inquiries can be directed to the corresponding author.

## Supplementary Materials

The following supporting information can be downloaded at https://www.mdpi.com/article/10.3390/antiox15070836/s1. Figure S1: The structural formula of the compound used. (A) The structural formula of Tanshinone IIA (Tan-IIA); Figure S2: The cytotoxicity of Tan-IIA in cultured NIH-3T3 and MRC-5 cells; Figure S3: Effects of Tan IIA on TGF-β1-induced mRNA and protein expression of Col-I, Col-III, α-SMA, and Nox4 in MRC-5 cells; Figure S4: Effects of Tan IIA on p-JNK/p-Jun and p-Smad2/3 and on fibrosis-related marker expression in MRC-5 cells.
