# Supplementary material for: Tanshinone IIA Attenuates Pulmonary Fibrosis via Dual Inhibition of JNK and Smad Signaling
Source: Antioxidants (Basel). 2026 Jul 2;15(7):836. doi: 10.3390/antiox15070836 (PMC13405772; doi:10.3390/antiox15070836)
Supplement: Supplementary file 1 [file antioxidants-15-00836-s001.zip › antioxidants-4326614-supplementary.pdf]

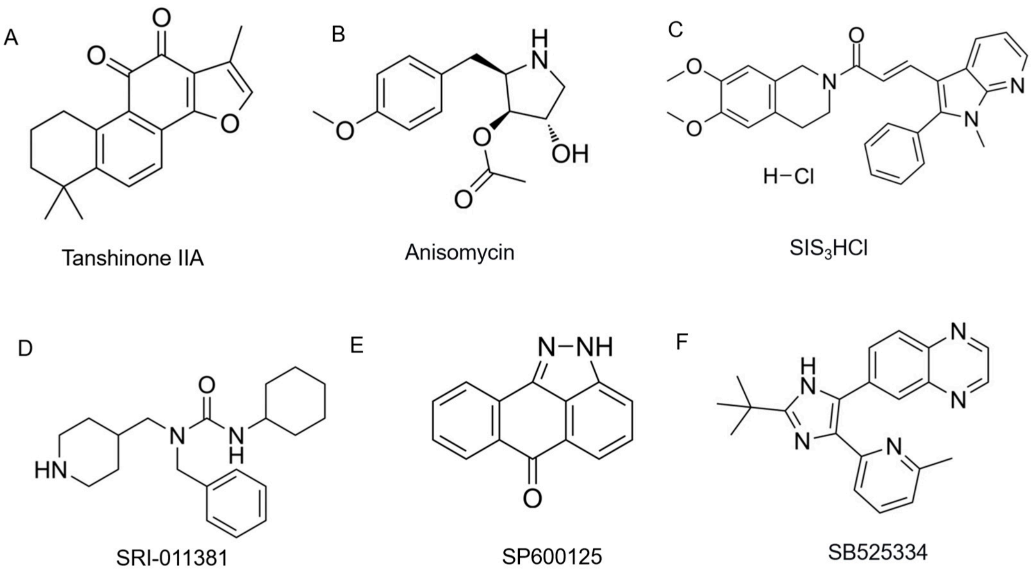

Supplementary Figure S1. The structural formula of the compound used. (A) The structural formula of Tanshinone IIA (Tan-ⅡA). (B) The structural formula of JNK activator Anisomycin. (C) The structural formula of Smad inhibitor SIS$_3$HCl. (D) The structural formula of Smad activator SRI-011381. (D) The structural formula of JNK inhibitor SP600125. (E) The structural formula of TGF-β1 inhibitor SB525334.

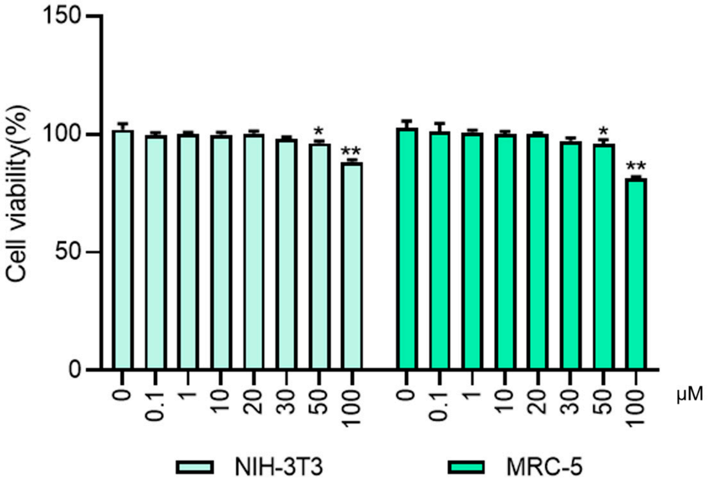

Supplementary Figure S2. The cytotoxicity of Tan-IIA in cultured NIH-3T3 and MRC-5 cells.

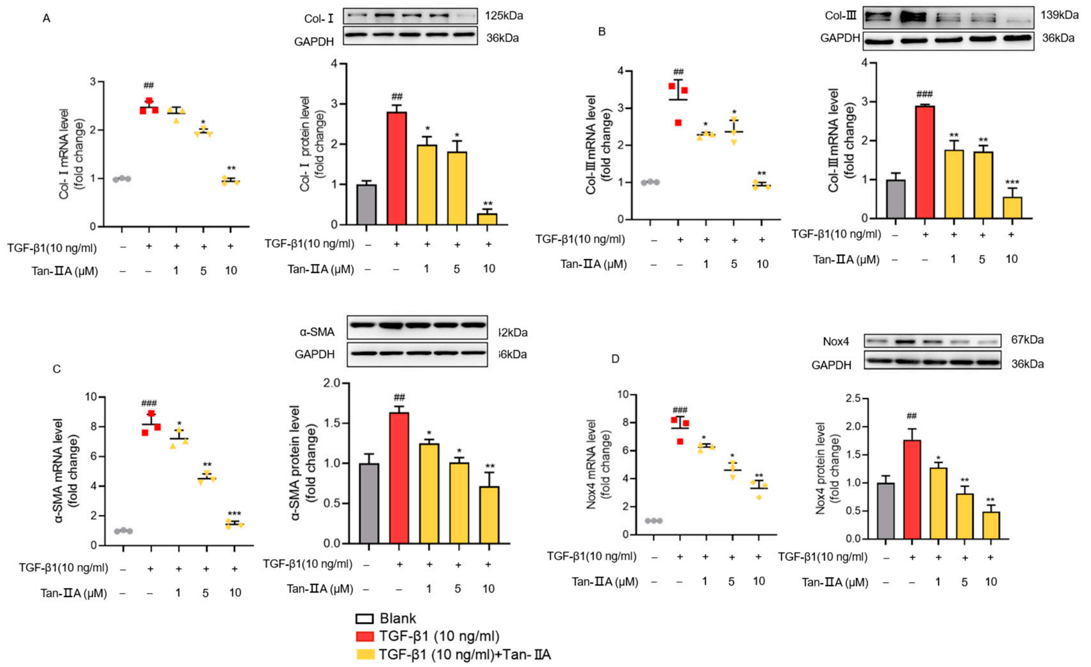

Supplementary Figure S3. Effects of Tan IIA on TGF-β1-induced mRNA and protein expression of Col-I, Col-III, α-SMA, and Nox4 in MRC-5 cells (A-D) The mRNA expression and the protein levels of Col-I, Col-III α-SMA and Nox4 in MRC-5 cells stimulated with TGF-β1 (10 ng/mL) and Tan-IIA. −/+ indicates absence or presence of compound treatment, respectively. Data are presented as the mean ± SD. Experiments were repeated three times. ## $p < 0.01$, ### $p < 0.001$ vs. Control; * $p < 0.05$, ** $p < 0.01$, *** $p < 0.001$ vs. TGF-β1.

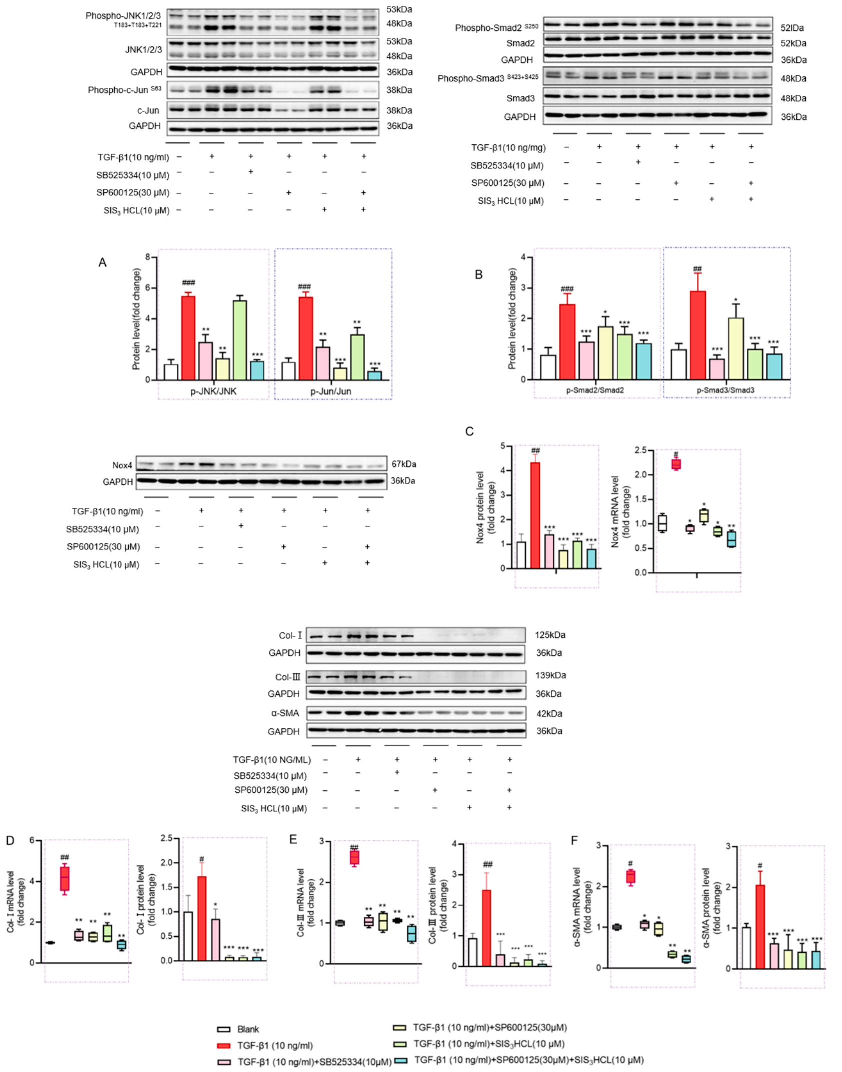

Supplementary Figure S4. Effects of Tan IIA on p-JNK/p-Jun and p-Smad2/3 and on fibrosis-related marker expression in MRC-5 cells (A) The protein levels of p-JNK/JNK and p-Jun/Jun in MRC-5 cells after drug treatment. (B) The protein levels of p-Smad2/Smad2 and p-Smad3/Smad3 in MRC-5 cells after drug treatment. (C-F) The mRNA levels (n = 4) and protein expression (n = 3) of Nox4, Col-I, Col-Ⅲ and α-SMA in MRC-5 cells after drug treatment. −/+ indicates absence or presence of compound treatment, respectively. Data were expressed as the mean ± SD. The experiment was repeated three times. Data were expressed as the mean ± SD. #$p < 0.05$, ##$p < 0.01$, ###$p < 0.001$ Vs Control; *$p < 0.05$, **$p < 0.01$, ***$p < 0.001$ vs TGF-β1
